# HIV-*Helicobacter pylori* Co-Infection: Antibiotic Resistance, Prevalence, and Risk Factors

**DOI:** 10.1371/journal.pone.0145119

**Published:** 2015-12-21

**Authors:** Marcel Nkuize, Stéphane De Wit, Vinciane Muls, Marc Delforge, Véronique Y. Miendje Deyi, Guy B. Cadière, Michel Buset

**Affiliations:** 1 Department of Gastroenterology and Hepatology, CHU Saint-Pierre, Université Libre de Bruxelles, Brussels, Belgium; 2 Division of Infectious Diseases, CHU Saint-Pierre, Université Libre de Bruxelles, Brussels, Belgium; 3 Department of Microbiology, CHU Brugmann, Université Libre de Bruxelles, Brussels, Belgium; 4 Department of Digestive Surgery, CHU Saint-Pierre, Université Libre de Bruxelles, Brussels, Belgium; University of Delhi, INDIA

## Abstract

**Background:**

Patients infected with human immunodeficiency virus (HIV) are living longer due to the availability of more potent treatments. However, prescription of antibiotics to treat or prevent infections in these patients may increase the likelihood of co-infection with antibiotic-resistant species.

**Aim:**

To compare antimicrobial susceptibility of *Helicobacter pylori (H*. *pylori)* in HIV-positive and HIV-negative patients and assess risk-factors for resistance.

**Methods:**

We prospectively collected data from consecutive HIV-positive and HIV-negative patients undergoing upper gastrointestinal endoscopy. Patients with *H*. *pylori*-positive gastric biopsies who had never received *H*. *pylori* treatment were included.

**Results:**

Of the 353 patients included, 93 were HIV-positive and 260 HIV-negative. Among the HIV-positive patients, 56 (60%) had been infected for <10 years, the median CD4+ count was 493 cells/μl and median viral load was 61 copies/mL; 66 (71%) were receiving antiretroviral therapy. HIV-positive patients were more often male (p = 0.009), had a lower body mass index (p<0.0001), and had less frequently received antibiotics during the 12-months prior to the endoscopy (p<0.0001) than HIV-negative patients. HIV-positive patients were more likely to have *H*. *pylori* resistant to levofloxacin (p = 0.0004), metronidazole (p = 0.01), or multiple antibiotics (p = 0.006). HIV-positive Black Africans were more likely to have resistant strains than were HIV-negative Black Africans (p = 0.04). Ethnicity and HIV status were independent risk factors for *H*. *pylori* resistance in all patients and acquired immune deficiency syndrome (AIDS) and sex were risk factors in HIV-positive patients.

**Conclusions:**

There was a higher prevalence of primary *H*. *pylori-*resistant strains in HIV-positive than in HIV-negative patients. AIDS and sex were predictors of *H*. *pylori* resistance in HIV-positive patients.

## Introduction

Human immunodeficiency virus (HIV) and *Helicobacter pylori (H*. *pylori*) infections are worldwide healthcare burdens. Improvements in antiretroviral treatment and patient care have increased the life expectancy of HIV-positive individuals [1]. As a consequence, HIV-positive patients suffering from *H*. *pylori* infection are seen more frequently than was the case before the advent of highly active antiretroviral therapy (HAART). We previously demonstrated increased *H*. *pylori* infection in HIV-positive patients who underwent upper gastrointestinal (UGI) endoscopy after the introduction of HAART compared to those in the pre-HAART era [2]. The prevalence of *H*. *pylori* infection among HIV-positive patients is variable, ranging from 10% to 76%, depending on the population studied and the geographic area [3, 4–7]. In a recent study in our center, the prevalence of *H*. *pylori* co-infection was 48/145 (33%) among symptomatic HIV-positive patients who underwent UGI endoscopy, and was more common in patients of Black African origin and in heterosexuals [3].

Complications related to *H*. *pylori* infection in the general population, such as gastro-duodenal ulcer and gastric carcinoma, have been extensively documented in different geographic regions. To avoid such complications, treatment to eradicate *H*. *pylori* is recommended [8]. Different therapeutic regimens have been recommended. The first-line treatment worldwide is triple therapy with a proton pump inhibitor (PPI) plus two antibiotics. In the general population, the success of triple therapy ranges from 85% to 90%. However, an increasing incidence of *H*. *pylori* treatment failure has been reported [9–11]. This has been attributed to various factors including patient compliance, polymorphism for PPI metabolism, and *H*. *pylori* susceptibility, which is the main factor affecting treatment outcome [8–13]. Increased antibiotic resistance has been described in various regions and has been attributed to high antibiotic consumption [13–15]. This finding may be particularly relevant for HIV-positive patients who are often exposed to antibiotics for chemoprevention of opportunistic diseases or treatment of acute infections.

Few studies have evaluated the impact of antibiotics on HIV-*H*. *pylori* co-infected patients. We, therefore, evaluated *H*. *pylori* susceptibility among HIV-positive and–negative patients seen at our center, and looked for predictive factors for *H*. *pylori* resistance.

## Materials and Methods

### Patients

This longitudinal observational study was carried out at CHU Saint-Pierre in Brussels, a general hospital that currently monitors close to 3000 HIV-positive patients on a regular basis and performs more than 2700 UGI endoscopies per annum.

We prospectively collected data on consecutive HIV-positive patients undergoing UGI endoscopy between 1st January 2008 and 31 December 2011. Patients with gastric biopsy samples positive for *H*. *pylori* infection on pathology and culture, and who had not received previous *H*. *pylori* treatment were eligible for inclusion in the study. During the same period, we collected data on a control group of consecutive HIV-negative patients undergoing UGI endoscopy prior to bariatric surgery for obesity who met the same criteria for *H*. *pylori* diagnosis and were naïve to *H*. *pylori* treatment. In our hospital, *H*. *pylori* eradication is mandatory before bariatric surgery (gastric bypass) for obesity.

### Methods

The study was conducted in conformity with the declaration of Helsinki and the protocol was approved by the local hospital ethics committee at CHU Saint-Pierre in Brussels. All procedures described in the study were performed for routine medical purposes. Written consent was obtained from all patients.

Parameters collected on the day of UGI endoscopy included demographics (age, sex, ethnicity, body mass index [BMI]), HIV status and parameters (duration of HIV infection, Center for Disease Control (CDC) stage, viral load, antiretroviral treatment, T-CD4 [CD4+] cell count), *Toxoplasma gondii* serology, closest to the date of endoscopy, and antibiotic use (including amoxicillin (AMX), clarithromycin (CLA), fluoroquinolones, tetracycline (TET), and metronidazole (MTZ)), *Toxoplasma gondii* or *Pneumocystis carinii* chemoprevention, or antimalarial drug use (including mefloquine, atovaquone, chloroquine, primaquine) within twelve months prior to endoscopy. Bismuth compounds are rarely used in Belgium, and were not recorded. Data were also collected on *H*. *pylori* treatment, including antibiotic susceptibility, type of treatment, tolerance, and response rate to first-line anti-*H*. *pylori* triple therapy.

#### Endoscopy and gastric sample biopsies

Patients underwent UGI endoscopy after fasting for 12 hours or overnight. At least eight gastric biopsy samples were taken (four from the angulus and four from the body). Four of the samples (two from each area) were immediately transferred to Amies Agar Gel Collection and Transport Swabs (Copan Diagnostic Inc., USA), and placed in a refrigerator at -5 to +5°C before being sent within 24 hours to the Department of Microbiology. The remaining samples were preserved in formaldehyde for histopathology.

#### *H*. *pylori* isolation and susceptibility testing

Upon receipt by the laboratory, gastric biopsy specimens were either frozen at -70°C until required, or processed immediately. Each specimen was homogenized in sterile water for 15 s and inoculated onto selective agar plates (in-house and Helicobacter agar Becton Dickinson, USA) by circular streaking with a bent pipette. Plates were incubated for 3 to 7 days at 37°C in a humid, microaerophilic incubator (Binder [serial no 08–51907], Germany) with a final atmosphere of 5% to 6% O_2_, 8% to 10% CO_2_, and 80% to 85% N_2_.

#### Antibiotic susceptibility

Susceptibility to MTZ, CLA, levofloxacin [LEV], AMX, and TET was assessed under routine conditions using disk diffusion methods (Neo-Sensitabs; Rosco, Taastrup, Denmark), and the minimum inhibitory concentration (MIC) determined by an agar dilution method. Isolates were classified as resistant with cut-off values of ≥1 mg/L for CLA, >8 mg/L for MTZ, ≥2 mg/L for AMX, >1 mg/L for LEV and ≥8 mg/L for TET [16, 17].

#### Pathology

Immunohistological staining was used to diagnose *H*. *pylori* infection. All slides were interpreted by the same pathologists according to the updated Sydney system scoring [18].

#### *H*. *pylori* treatment regimen

Depending on the antibiotic susceptibility, patients received one of the following regimens:

- triple therapy: a PPI (omeprazole 20 mg, esomeprazole 40 mg, or pantoprazole 40 mg) plus two antibiotics (AMX 1000 mg, CLA 500 mg, TET 500 mg, or MTZ 500 mg) twice daily for 10 days (range 7 to 14 days)
- sequential therapy: a PPI plus amoxicillin 1000 mg twice daily for 5 days followed by a PPI plus clarithromycin 500 mg and metronidazole 500 mg twice daily for 5 days
- quadruple therapy: a PPI twice daily, TET-HCL 500 mg plus colloidal bismuth subcitrate 500 mg four times daily, and metronidazole 500 mg three times daily for 10 days.

#### Urea breath test (UBT)

A standard UBT protocol was performed after overnight fasting. Briefly, an exhaled breath sample was obtained before (T0), and at 10 (T1) and 15 (T2) minutes after administration of two microcuries of ^14^C-urea liquid mixed with 30 ml of water given orally. Samples T0, T1, and T2 were analyzed using a PerkinElmer Tricarb-1500 scintillation counter. The final result was obtained using the formula: [T1 minus T0, divided by the value provided by the scintillation counter for a 1/1000 dilution of 2 microcuries standard dose] multiplied by the weight (in kilograms). The T2 value was used as a control. A value <1.5 was defined as negative, indicating eradication of *H*. *pylori* infection [19].

The test was performed five to eight weeks after the end of the *H*. *pylori* treatment regimen. To insure correct interpretation of the results, patients were asked to confirm that no other antibiotic or PPI treatment had been administered since completion of *H*. *pylori* therapy.

#### Statistics

Patients were classified according to HIV status (positive or negative) and data from these two groups were compared. Descriptive statistics are expressed using mean values and range for quantitative measurements and percentages for qualitative measurements. Fisher’s exact test and the chi-square test were used for comparisons between groups. Between-group differences are presented as the difference between the means of each group with the 95% confidence interval (95% CI) for each comparison. A crude odds ratio (OR) with its 95% CI is presented for each comparison.

Predictors of *H*. *pylori* resistance were assessed in all patients using the chi-square test and were confirmed by logistic regression in multivariable analysis. Parameters used for the study of predictors of resistance in this group included age, sex, ethnicity, and HIV status.

Predictors of *H*. *pylori* resistance were assessed in HIV-positive patients using the chi-square test and were confirmed by logistic regression in multivariable analysis for any parameter with a p-value ≤0.1 in univariate analysis. Parameters used for the study of predictors of resistance in this group included sex, ethnicity, HIV viral load and duration of HIV infection, antiretroviral treatment, and a diagnosis of acquired immunodeficiency syndrome (AIDS).

Analyses were performed using SAS statistical software (version 9·2; SAS Institute, Cary, NC, USA). A p value of < 0.05 was considered to be significant.

## Results

**Fig 1:**
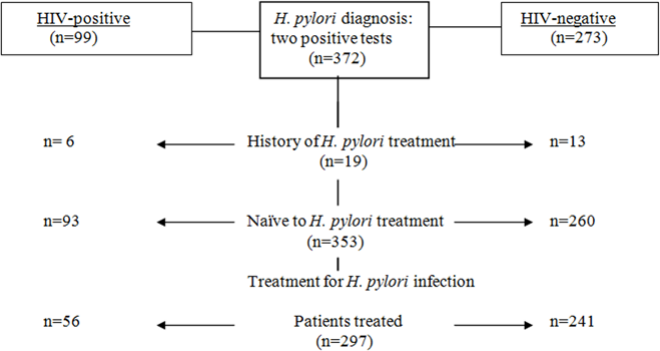
Study design.

Of 372 patients with positive tests for *H*. *pylori* (99 HIV-positive and 273 HIV-negative), 353 (93 HIV-positive and 260 HIV-negative) were naïve to *H*. *pylori* treatment and were included in the study (Fig 1). Among the HIV-infected patients, 31 (44%) had a diagnosis of AIDS and 66 (71.0%) were receiving antiretroviral therapy. The median CD4 count was 489 cells/μl, but was lower in Black Africans and North Africans than in Caucasian Europeans (p = 0.03). *Toxoplasma gondii* (*T*. *gondii*) IgG antibodies were present in 43/80 (53.1%) patients screened; 20/93 (21%) patients were receiving trimethoprim-sulfamethoxazole for chemoprevention against *T*. *gondii* or *Pneumocystosis carnii* or for an acute infection.

Compared to HIV-negative patients, HIV-positive patients were more often male (p = 0.009), had a lower BMI (p<0.0001), and had less frequently received antibiotics during the 12 months prior to UGI endoscopy (p<0.0001) (Table 1). In the HIV-positive patients, those with AIDS were more likely to have received antibiotics during the 12 months prior to UGI endoscopy than those without AIDS (7/31 [22.5%] vs. 3/62 [4.8%]) OR [95%CI] 0.17 [0.04–0.73], p = 0.009). Anti-malarial drug use and mean age were similar in the HIV-positive and -negative groups. The majority of HIV-positive patients were black African. Among HIV-negative patients, most were Caucasian Europeans.

**Table 1:** Characteristics of included patients.

Patient characteristics	HIV-positive (n = 93)	HIV-negative (n = 260)	OR, 95% CI	P-value
**Mean age(range), years**	40.9 (21–75)	39.6 (19–76)	-	0.5
**Female**	41 (44.0%)	155 (59.6%)	0.53 (0.33–0.86)	0.009
**Ethnicity**				
Black African	54 (58.0%)	57 (21.9%)		
North African	9 (9.6%)	94 (36.1%)		
White European/Other	27 (29.0%)/3 (3.2%)	97 (37.3%) /12 (4.6%)		
**Body Mass Index (kg/m** ^**2**^ **), median (Q1-Q3)**	24.5 (22–28.5)	41 (39–45)		<0.0001
**Medication used in the previous 12 months**	n = 89	n = 210		
Amoxicillin/clarithromycin/ metronidazole/tetracycline	10/89 (11.2%)	74/210 (35.2%)	0.23 (0.11–0.47)	<0.0001
antimalaria chemoprevention	4 (4.4%)	6 (2.8%)	0.52 (0.14–1.90)	0.3
antimalaria chemotherapy	0	0		
Proton pump inhibitor or H2 blockers	12/89 (13.4%)	109/206 (52.9%)		<0.0001
***Specific characteristics of HIV-positive patients***				
**Diagnosis of AIDS**	31 (44%)	-		
**Duration of HIV infection, years (Q1-Q3)**	6.4 (1.2–11.8)	-		
**Duration of HIV infection > 10 years**	56 (60.2%)	-		
**Median CD4 count, cells/μl (Q1-Q3)**	489 (334–648)*	-		
**Median viral load, copies/ml (Q1-Q3)**	51 (50–4x10^4^)	-		
**CDC stage**		-		
B	9 (9.7%)			
C	31 (33.3%)			
Neither B nor C	53 (57.0%)			
**Seropositive for *T*. *gondii* IgG abs**	43/80 (53.7%)**	-		
**Receiving TMP-SFX**	20 (21.5%)	-		
**Receiving antiretroviral therapy**	66 (71.0%)	-		

OR: odds ratio, 95% CI: 95% confidence interval, Q1-Q3: 1^st^ and 3^rd^ quartile, *T*. *gondii* IgG abs: *Toxoplasma gondii* IgG antibodies

* Black Africans vs. North Africans vs. Caucasian Europeans: 435.0 [271.0–599.0] vs. 549.0 [357.0–720.0] vs. 549.5 [386.0–822.0], p = 0.03)

**13 patients had unknown *Toxoplasma gondii* serology at the time of the UGI endoscopy, TMP-SFX trimethoprim-sulfamethoxazole

Overall, *H*. *pylori* resistance was present in 199 (56.3%) patients. Resistance to MTZ was observed most frequently, followed by resistance to LEV and then CLA (Table 2). *H*. *pylori* resistance to LEV (p = 0.0004), MTZ (p = 0.01), or multiple antibiotics was more frequent in HIV-positive than in HIV-negative patients. No *H*. *pylori* strains were resistant to AMX or TET in either group. Resistance to CLA was more frequent in patients with a CD4 count < 200 cells/μL than in those with a CD4 count ≥ 200 cells/μL (3/7 (42.8%) vs. 10/84 (11.9%), p = 0.05).

**Table 2:** Summary of primary resistance of *H*. *pylori* in HIV-positive and HIV-negative patients.

Resistance	ALL (n = 353)	HIV-positive (n = 93)^†^	HIV-negative (n = 260)	OR, 95%CI	P-value
***All resistance***	199 (56.3%)	65 (69.8%)	134 (51.5%)	2.18 (1.31–3.61)	0.002
***Single resistance***					
**AMX**	0	0	0	-	-
CLA*	40/353 (11.3%)	13/93 (13.9%)	27/260 (10.3%)	0.7 (0.35–1.44)	0.34
**LEV**	76/353 (21.5%)	32/93 (34.4%)	44/260 (16.9%)	2.5 (1.50–4.40)	0.0004
**MTZ#**	146/350 (41.7%)	48/92 (52.1%)	98/258 (37.9%)	1.7 (1.10–2.87)	0.01
**TET**	0	0	0	-	-
***≥ 2 resistances***	59 (16.7%)	24 (25.8%)**	35 (13.4%)***	0.44 (0.24–0.80)	0.006
**CLA-MTZ**	14/353 (3.9%)	9/93 (9.6%)	5/260 (1.9%)	5.46 (1.78–16.75)	0.002
**CLA-LEV**	16/353 (4.5%)	5/93 (5.3%)	11/260 (4.2%)	1.28 (0.43–3.80)	0.6
**LEV-MTZ**	37/353 (10.4%)	18/93 (19.3%)	19/260 (7.3%)	3.04 (1.51–6.09)	0.002
**CLA-MTZ-LEV**	4/353 (1.1%)	4/93 (4.3%)	0/260 (0.0%)	0.25 (0.21–0.30)	0.004

OR: odds ratio, 95% CI: 95% confidence interval, AMX: amoxicillin, CLA: clarithromycin, LEV: levoflaxacin, MTZ: metronidazole, TET: tetracycline

† 3 of 28 (10.7%) patients without *H*. *pylori*-resistant strains and 17 of 65 (26.5%) patients with *H*. *pylori*-resistant strains were receiving trimethoprim-sulfathoxazole (OR 2.95 (0.78–11.03), p = 0.1).

# tree strain death (1 and 2 among HIV-positive and -negative patients, respectively)

** of 24 patients, 18 were Black Africans, 5 were white Europeans, and 1 was from another ethnicity

*** of 35 patients, 8 were Black Africans, 9 were North Africans, 15 were white Europeans, and 3 were from other ethnicities.

Black Africans who were HIV-positive had a significantly greater incidence of resistant strains than did Black Africans who were HIV-negative (p = 0.04, Table 3). Within the other ethnicities, HIV-positive patients were more likely to have resistant strains than HIV-negative patients, but the differences were not statistically significant. HIV-positive women were more likely to have resistant strains than HIV-negative women (p = 0.001), notably for LEV (p = 0.001) and MTZ (p = 0.03). There were no significant differences in the incidences of resistance in male HIV-positive and -negative patients.

**Table 3:** Prevalence of *H*. *pylori* resistant strains within each ethnic group and sex, stratified according to HIV status.

*H*. *pylori* primary resistance				
Parameter	HIV-positive	HIV-negative	OR, 95% CI	P-value
**Ethnicity**				
**Black Africans (n = 54/57)**	**41 (75.9%)**	**33 (57.8%)**	**2.2 (1.01–5.18)**	**0.04**
**CLA-R**	7 (12.9%)	2 (3.5%)	4.09 (0.81–20.6)	0.08
**LEV-R**	24 (44.4%)	11 (19.3%)	3.34 (1.43–7.81)	0.004
**MTZ-R**	32 (59.2%)	28 (49.1)	1.50 (0.71–3.19)	0.3
**North Africans (n = 9/94)**	**5 (55.5%)**	**39 (41.4%)**	**1.76 (0.44–6.98)**	**0.5**
**CLA-R**	1 (11%)	4 (4.2%)	2.8 (0.27–28)	0.3
**LEV-R**	0	13 (13.8%)	1.16 (1.07–1.25)	0.5
**MTZ-R**	4/8* (50%)	31/93* (33.3%)	2.0 (0.46–8.53)	0.4
**White Europeans (n = 27/97)**	**17/27(62.9%)**	**51/97 (52.5%)**	**1.5 (0.63–3.68)**	**0.5**
**CLA-R**	5 (18.5%)	20 (20.6%)	0.87 (0.29–2.59)	1
**LEV-R**	7 (25.9%)	15 (15.4%)	1.91 (0.68–5.31)	0.2
**MTZ-R**	10 (37.0%)	31 (32.2%)	1.23 (0.50–3.00)	0.6
**Sex**				
**Female (F)**	33/41 (80.4%)	81/155 (52.2%)	3.7 (1.63–8.67)	0.001
**Male (M)**	32/52 (61.5%)	53/105 (50.4%)	1.5 (0.79–3.08)	0.2
**Clarithromycin-resistance**				
**F**	5/41 (12.2%)	19/155 (12.2%)	0.9 (0.34–2.84)	1
**M**	8/52 (15.3%)	8/105 (7.6%)	2.2 (0.77–6.25)	0.1
**Levofloxacin-resistance**				
**F**	17/41 (41.4%)	26/155 (16.7%)	3.5 (1.65–7.44)	0.001
**M**	15/52 (28.8%)	18/105 (17.1%)	1.95 (0.89–4.29)	0.09
**Metronidazole-resistance**				
**F**	24/41 (58.5%)	61/154 (39.6%)	2.1 (1.06–4.33)	0.03
**M**	24/51 (47.0%)	37/104 (35.5%)	1.6 (0.81–3.17)	0.2

OR: odds ratio, CI: confidence interval, CLA: clarithromycin, R: resistance, LEV: levofloxacin, MTZ: metronidazole

* one patient with this strain died

In a sub-analysis, the prevalence of *H*. *pylori* resistant strains was assessed for each antibiotic according to HIV status and then stratified according to ethnic group. These analyses showed that resistant strains were significantly more common among Black African patients than among North African and Caucasian Europeans (74/111 [66.6%] vs. 44/103 [42.7%] vs. 68/124 [54.8%], p = 0.002), notably for CLA (p = 0.0002), LEV (p = 0.002) and MTZ (p = 0.002). With regard to HIV status, strains resistant to LEV or MTZ were more common among HIV-positive Black Africans than among other ethnicities (p = 0.01 and 0.08, respectively), whereas strains resistant to CLA were more frequent among HIV-negative Caucasian Europeans than among other ethnicities (p = 0.0002).

In multivariable analysis in the whole cohort, ethnicity and HIV status were independent predictors of *H*. *pylori* resistance (Table 4). In the subgroup of HIV-infected patients, sex and AIDS were independent predictors of *H*. *pylori* resistance (Table 5).

**Table 4:** Unadjusted and adjusted analysis of risk factors for carrying *H*. *pylori* resistant strains in all patients.

Unadjusted				Adjusted	
Parameters	Resistance		P-value	OR, 95% CI	P-value
	No	Yes			
**Sex**			0.4	-	
Women	82 (41.8%)	114 (58.1%)			
Men	72 (45.8%)	85 (54.1%)			
**Ethnicity**			0.002		0.04
WE	56 (45.1%)	68 (54.8%)		Reference	
BA	37 (33.3%)	74 (66.6%)		1.40 (0.81–2.43)	
NA	59 (57.2%)	44 (42.7%)		0.53 (0.30–0.91)	
**Age**			0.3		
< 40	46 (49.5%)	47 (50.5%)			
40–64	107 (41.8%)	149 (58.2%)			
≥ 65	1 (0.7%)	3 (1.5%)			
**HIV status**			0.002	0.53** (0.30–0.91)	0.02
Negative	126 (48.4%)	134 (51.5%)			
Positive	28 (30.1%)	65 (69.8%)			

OR: odds ratio, CI: confidence interval, WE: white European, BA: Black African, NA: North African

** HIV-negative vs. HIV-positive

**Table 5:** Unadjusted and adjusted analysis of risk factors for carrying *H*. *pylori* resistant strains in HIV-positive patients.

	Unadjusted		Adjusted	
Parameters	Resistance:yes	P-value	OR 95% CI	P-value
**Sex**		0.04	3.67 (1.24–10.86)	0.01
F (n = 41)	33 (80.4%)			
M (n = 52)	32 (61.5%)			
**Ethnicity**		0.3		
BA (n = 54)	40 (74.07%)			
non-BA (n = 39)	25 (64.10%)			
**HIV viral load**		0.7		
< 50 (n = 22)	16 (72.7%)			
≥ 50 (n = 67)	47 (70.1%)			
**Duration of HIV infection**		0.07	1.98 (0.73–5.36)	0.17
< 10 years (n = 56)	43 (76.7%)			
≥ 10 years (n = 37)	22 (59.4%)			
**Antiretroviral therapy**		0.6		
No (n = 27)	20 (74.0%)			
Yes (n = 66)	45 (68.1%)			
**AIDS**		0.1	3.82 (1.11–13.08)	0.03
No (n = 62)	40 (64.5%)			
Yes (n = 31)	25 (80.6%)			

CI: confidence interval

*H*. *pylori* treatment was administered to 62/93 HIV-positive and 241/260 HIV-negative patients. The remaining 31 HIV-positive patients were not treated because of poor follow-up (n = 20), worsening medical condition (n = 8), consent withdrawal (n = 3); 19 HIV-negative patients were lost to follow-up. First-line therapy for *H*. *pylori* was most commonly triple therapy in 50/62 (80.6%) and 214/241 (88.7%) HIV-positive and -negative patients, respectively. Other treatments were sequential therapy (7 [11.2%] vs. 24 [9.9%]), and quadruple therapy (5 [8.0%] vs. 3 [1.2%]) in HIV-positive and -negative patients, respectively. Treatment was tolerated equally well in both groups. A UBT was performed five to eight weeks (mean 6 weeks) after cessation of the *H*. *pylori* treatment in a subset of patients: 62 and 241 patients from the HIV-positive and -negative groups, respectively. Only the triple therapy response is given. The UBT showed a response rate to triple therapy of 40/50 (80.0%) and 179/214 (83.6%) in HIV-positive and HIV-negative patients, respectively (p = 0.5). The response rate was 28/37 (75.6%) among antiretroviral-treated patients and 12/13 (92.3%) among antiretroviral-untreated patients (OR and 95% confidence interval: 0.25 (0.02–2.27), p = 0.2).

## Discussion

To our knowledge, this is the first prospective study in which the susceptibility of *H*. *pylori* strains to antibiotics has been compared in HIV-positive and HIV-negative patients [14]. Overall, resistance to MTZ was most frequent, followed by LEV and CLA resistance. Our results are similar to those of a previous multicenter study carried out among unselected patients in Belgium, but differ from studies conducted in Africa, where a much higher prevalence of resistant strains has been found [17, 20, 21]. Our major finding was the significantly greater prevalence of primary *H*. *pylori* resistance to MTZ and LEV in HIV-positive patients compared to HIV-negative patients, and a trend towards a greater prevalence of CLA resistance.

In multivariable analysis, HIV status was a strong and independent predictive factor for resistance of *H*. *pylori*. It is possible that the higher prevalence of *H*. *pylori* resistance in HIV-positive patients could be due to greater previous cumulative antibiotic exposure, either for treatment of acute infections or for chemoprevention of opportunistic diseases. However, except for the patients with AIDS, HIV-positive patients in this study had less frequently received antibiotics in the 12 months prior to *H*. *pylori* diagnosis than had the HIV-negative patients. It has been suggested that twice as many *Clostridium difficile* infections (often treated with MTZ) occur in HIV-positive patients (notably with AIDS or lower T CD4 counts or post-antibiotic exposure) than in HIV-negative patients [22, 23]. However, we were unable to determine whether this was the case in these patients. HIV-positive patients also had a higher prevalence of *H*. *pylori* resistant to LEV than HIV-negative patients; this has not been documented previously. Fluoroquinolone use may be higher in HIV-positive than in HIV-negative patients because of its use as empiric treatment for infectious diarrhea, urinary tract infection, or pneumonia (25-fold), which are more common in HIV-positive patients [24, 25]. Finally, the prevalence of *H*. *pylori* CLA resistance was nearly 14% in HIV-positive patients compared to 10% in HIV-negative patients. The frequency of *H*. *pylori* CLA resistance in HIV-positive patients was higher than that recently found among unselected patients in Belgium [17]. This greater prevalence could be, at least in part, due to the use of macrolides in the chemoprevention of Mycobacterium avium complex (MAC) infection in severely immunocompromised HIV patients (CD4 counts <100). Interestingly, we found that CLA resistance was more frequent in HIV-positive patients with CD4 counts <200 cells/μL than in those with higher CD4 counts. Use of macrolides for other indications, including respiratory infections with atypical microorganisms and sexually transmitted diseases such as chlamydiosis, may also be involved [1, 26]. HIV-positive patients had more multidrug-resistant *H*. *pylori* strains than did HIV-negative patients.

Interestingly, AIDS was an independent predictive factor for carrying *H*. *pylori* resistant strains. This finding has not been published previously. The explanation could be that, as we have demonstrated, patients with AIDS were exposed to more antibiotics, which could potentially lead to *H*. *pylori* resistance. Moreover, in our hospital, 20% of patients newly diagnosed with HIV infection have a CD4+ count < 200 and are likely to receive antibiotics to cure or prevent infection (S. De Wit, personal communication).

Another independent risk factor for *H*. *pylori* resistance was ethnicity. Indeed, we demonstrated that the prevalence of resistant strains was significantly different across ethnicities. Resistant *H*. *pylori* strains were more frequently isolated from patients of Black African ethnicity than from those of North African or Caucasian European ethnicities. This was particularly true for MTZ, LEV, and multidrug resistance in Black African patients who were HIV-positive. The frequent use of imidazole derivatives for the treatment of gynecological or protozoal infections in Africa, the region from which these patients originated, may play a role in this increased MTZ resistance. Alternatively, a subset of Black African patients may have been infected with *H*. *pylori* in Africa (an endemic region for *H*. *pylori)* with a strain that was MTZ resistant without previous exposure to this drug [20, 21]. However, this explanation is less likely because the prevalence of MTZ resistance among Black Africans in our study was less than that found in some African regions in which virtually all patients are infected with MTZ-resistant strains [15, 20, 21]. The prevalence of *H*. *pylori* strains resistant to LEV was higher among HIV-infected Black Africans than among HIV-infected patients from other ethnicities. It is possible that Black African HIV-positive patients may have been exposed more commonly to LEV for appropriate indications, such as those already mentioned above (urinary or digestive infections) because they had more pronounced immune depression (lower CD4 counts) and a higher risk of pneumonia, or they may have been exposed to inappropriate prescription of fluoroquinolones [15, 20, 21, 24, 25].

Overall, multidrug-resistant strains were more common among patients of Black African ethnicity than North African and Caucasian European ethnicities. This was particularly true among HIV-positive black African patients [17, 20, 21]. HIV-positive Black Africans may have had more cumulative antibiotic exposures than other ethnicities given that they had significantly lower median CD4 counts.

Sex was also an independent risk factor for *H*. *pylori* resistance among HIV-positive patients in contrast to the whole cohort. HIV-infected females were more commonly infected with *H*. *pylori* strains resistant to LEV and MTZ. Female sex is associated with more frequent urinary infections and gynecologic infections, which are often treated with LEV and MTZ, respectively. This is an additional risk factor that could be exacerbated in the presence of HIV/AIDS. Sex has been reported as a risk factor for resistance in other studies in the general population [14, 17, 27].

Another interesting finding is the absence of *H*. *pylori* with resistance to AMX or TET in all patients enrolled. The absence of *H*. *pylori* resistance to AMX or TET was also reported in a multicenter study of unselected patients from Belgium [17]. In contrast, *H*. *pylori* strains resistant to AMX and TET have been described in Africa [15, 20, 21].

Overall, the most commonly prescribed treatment regimen was triple therapy. HIV-positive and HIV-negative patients in our study achieved lower eradication rates compared to previous reports, despite good tolerance [13]. The end-point of this study was not the effectiveness of anti-*H*. *pylori* treatment, so we cannot draw any definitive conclusions on this aspect from our results. Nevertheless, we observed a lower response rate among HIV-positive patients treated with antiretroviral drugs compared to HIV-positive patients who were not treated with antiretrovirals; it may, therefore, be interesting to evaluate the role of higher CD4 count (a risk-factor for *H*. *pylori* infection) on response rates as well as the possibility of an interaction between anti-*H*. *pylori* and antiretroviral drugs on treatment response [2,28]. Several arguments have emerged from the current literature that may explain, in part, the suboptimal response to anti-*H*. *pylori* treatment seen in our patients [8–13, 29–32]. Antibiotic-resistance is the major cause of failure to eradicate *H*. *pylori*. CLA-resistant strains are, for example, associated with a 70% reduction in efficacy of *H*. *pylori* triple therapy. There was a high prevalence of *H*. *pylori* CLA-resistant strains among HIV-positive patients, nearly reaching the threshold of 15 to 20% at which the use of this drug in the treatment against *H*. *pylori* should be avoided [8]. There was also variation in the duration of triple therapy in this study, ranging from 7 to 14 days, and duration of treatment has been shown to impact on the effectiveness of triple therapy against *H*. *pylori* [8–13].

The strengths of this study include the prospective collection of data, and the enrollment of a large number of patients who were all naïve to *H*. *pylori* treatment and had antibiotic susceptibility results available. Moreover, patients were well-matched for age and were from different ethnicities. The study provides insight into the chemosusceptibility of *H*. *pylori* strains among subpopulations, notably those who were HIV-positive. Limitations of this study include the fact that the HIV-positive North African group was underrepresented. However, this pattern reflects the current epidemiological trend in our hospital. In addition, treatment for *H*. *pylori* infection was not studied extensively because effectiveness of treatment was not the end-point.

## Conclusion

The data presented in this paper demonstrate that there is a higher prevalence of primary *H*. *pylori* resistance in HIV-positive compared to HIV-negative patients. In these patients, primary resistance was most frequently to CLA, LEV, and MTZ. Multidrug resistance was also frequently found, notably among Black Africans. Among HIV-infected patients, sex and a diagnosis of AIDS were independent predictors of *H*. *pylori* resistance.

A careful history of antimicrobial use should be taken and results of susceptibility testing awaited in HIV-infected patients and individuals from developing countries before anti-*H*. *pylori* therapy is started. If antimicrobial susceptibility testing is not available, we suggest taking into account the patient’s risk profile for resistance and avoiding regimens that include clarithromycin or fluoroquinolones. Overall, these data provide evidence that the assessment of *H*. *pylori* drug resistance is warranted in HIV-positive patients and further studies in other populations/regions are desirable.

## References

[1] Palella FJ Jr, Delaney KM, Moorman AC, Loveless MO, Fuhrer J, Satten GA, et al Declining morbidity and mortality among patients with advanced human immunodeficiency virus infection. N Engl J Med.1998;338:853–860. 9516219 10.1056/NEJM199803263381301

[2] Nkuize M, Dewit S, Muls V, Arvanitakis M, Buset M. Upper gastrointestinal endoscopic findings in the era of highly active antiretroviral therapy. HIV Med. 2010;11:412–417. 10.1111/j.1468-1293.2009.00807.x 20146733

[3] Nkuize M, Dewit S, Muls V, Ntounda R, Gomez-Galdon M, Buset M. Comparison of demographic characteristics and upper gastrointestinal endoscopy findings in HIV-positive, antiretroviral-treated patients with and without *Helicobacter pylori* coinfection. Helicobacter. 2012;17:153–159. 10.1111/j.1523-5378.2011.00929.x 22404447

[4] Marano BJ, Smith F, Bonanno CA. Helicobacter pylori prevalence in acquired immunodeficiency syndrome. Am J Gastroenterol.1993;88:687–690. 8480733

[5] Alimohamed F, Lule GN, Nyong’o A, Bwayo J, Rana FS. Prevalence of *Helicobacter pylori* and endoscopic findings in HIV seropositive patients with upper gastrointestinal tract symptoms at Kenyatta national hospital, Nairobi. East Afr Med J. 2002;79:226–231. 12638804 10.4314/eamj.v79i5.8858

[6] Chiu HM, Wu MS, Hung CC, Shun CT, Lin JT. Low prevalence of *Helicobacter pylori* but high prevalence of cytomegalovirus-associated peptic ulcer disease in AIDS patients: comparative study of asymptomatic subjects evaluated by endoscopy and CD4 counts. J Gastroenterol Hepatol.2004;19:423–428. 15012780 10.1111/j.1440-1746.2003.03278.x

[7] Fialho ABC, Braga-Neto MB, Guerra AJC, Fialho AM, Fernandes KC, Sun JL, et al Low prevalence of *H*. *pylori* infection in HIV-positive patients in the Northeast of Brazil. BMC Gastroenterol.2011;11:13 10.1186/1471-230X-11-13 21333017 PMC3055236

[8] Malfertheiner P, Megraud F, O’Morain CA, Atherton J, Axon AT, Bazzoli F, et al Management of *Helicobacter pylori* infection-the Maastricht IV/Florence Consensus report. Gut. 2012;61:646–664. 10.1136/gutjnl-2012-302084 22491499

[9] Sachs G, Scott DR, Wen Y. Gastric infection by *Helicobacter pylori* . Curr Gastroenterol Rep.2011;13:540–546. 10.1007/s11894-011-0226-4 21993716 PMC4531091

[10] Sachs G, Wen Y, Scott DR. Gastric infection by *Helicobacter pylori* . Curr Gastroenterol Rep.2009;11:455–461. 19903421 10.1007/s11894-009-0070-y PMC4492511

[11] Graham DY, Fischbach L. *Helicobacter pylori* treatment in the era of increasing antibiotic resistance. Gut.2010;59:1143–1153. 10.1136/gut.2009.192757 20525969

[12] Furuta T, Shirai N, Takashima M, Xiao F, Hanai H, Sugimura H, et al Effect of genotypic differences in CYP2C19 on cure rates for Helicobacter pylori infection by triple therapy with a proton pump inhibitor, amoxicillin, and clarithromycin. Clin Pharmacol Ther. 2001;69:158–168. 11240980 10.1067/mcp.2001.113959

[13] Graham DY, Schiotani A. New concepts of resistance in the treatment of *Helicobacter pylori* infection. Nat Clin Pract Gastroenterol Hepatol. 2008;5:321–331. 10.1038/ncpgasthep1138 18446147 PMC2841357

[14] Megraud F, Coenen S, Versporten S, Kist M, Lopez-Brea M, Hirschl AM, et al *Helicobacter pylori* resistance to antibiotics in Europe and its relationship to antibiotic consumption. Gut. 2013;62:34–42. 10.1136/gutjnl-2012-302254 22580412

[15] De Francesco V, Giorgio F, Hassan C, Manes G, Vannella L, Panella C, et al Worldwide *H*. *pylori* antibiotic resistance: a systematic review. J Gastrointestin Liver Dis. 2010;19:409–414. 21188333

[16] Clinical and Laboratory Standards Institute. Performance standards for antimicrobial susceptibility testing: 19th informational supplement. Clinical and Laboratory Standards Institute, Wayne, PA 2009; vol. 29, no. 3. M100-S19.

[17] Miendje Deyi VY, Bontems P, Vanderpas J, De Koster E, Ntounda R, Van den Borre C, et al Routine survey determinations of resistance of *Helicobacter pylori* to antimicrobials over the last 20 years (1999 to 2009) in Belgium. J Clin Microbiol. 2011; 49: 2200–2209. 10.1128/JCM.02642-10 21450969 PMC3122765

[18] Dixon MF, Genta RM, Yardley JH, Correa P. participants in the international workshop on the histopathology of gastritis, Houston 1994. Classification and grading of gastritis–The updated Sydney System. Am J Surg Pathol. 1966;20:1161–1181. 10.1097/00000478-199610000-00001 8827022

[19] Desroches JJ, Lahaie RG, Picard M, Morais J, Dumont A, Gaudreau C, et al Methodological validation and clinical usefulness of carbon-14-urea breath test for documentation of the presence and eradication of *Helicobacter pylori* infection. J Nucl Med. 1997;38:1141–1145. 9225808

[20] Ndip RN, Malange Takam AE, Ojongokpoko JEA, Luma HN, Malongue A, Akoachere JF, et al *Helicobacter pylori* isolates recovered from gastric biopsies of patients with gastro-duodenal pathologies in Cameroon: current status of antibiogram. Trop Med Int Health. 2008;13:848–854. 10.1111/j.1365-3156.2008.02062.x 18384477

[21] Tanih NF, Okeleye BI, Naidoo N, Clarke AM, Mkwetshana N, Green E, et al Marked susceptibility of South African *Helicobacter pylori* strains to ciprofloxacin and amoxicillin: Clinical implications. S Afr J Med. 2010;100:49–52. 20429489

[22] Collini JP, Bauer M, Kuijper EJ, Dockrell DH. *Clostridium difficile* infection in HIV-seropositive individuals and transplant recipients. J Infect. 2012;64:131–147. 10.1016/j.jinf.2011.12.003 22178989

[23] Collini JP, Bauer M, Dockrell DH. *Clostridium difficile* infection in patients with HIV/AIDS. Curr HIV/AIDS rep. 2013;10: 273–282. 10.1007/s11904-013-0162-z 23657793

[24] Feikin DR, Feldman C, Schuchat A, Janoff EN. Global strategies to prevent bacterial pneumonia in adults with HIV disease. Lancet Infect Dis. 2004;4:445–455. 15219555 10.1016/S1473-3099(04)01060-6

[25] Morris AM, Huang L, Bacchetti P, Turner J, Hopewell PC, Wallace JM, et al Permanent declines in pulmonary function following pneumonia in human immunodeficiency virus-infected persons. The Pulmonary Complications of HIV Infection Study Group. Am J Respir Crit Care Med. 2000;162:612–616. 10934095 10.1164/ajrccm.162.2.9912058

[26] Zuckerman JM. Macrolides and ketolides: azithromycin, clarithromycin, telithromycin. Infect Dis Clin N Am. 2004;18:621–649. 10.1016/j.idc.2004.04.010 15308279

[27] Haggerty CL, Ness RB. Newest approaches to treatment of pelvic inflammatory disease: a review of recent randomized clinical trials. Clin Infect Dis. 2007;44:953–960. 17342647 10.1086/512191

[28] Nachega JB, Hsu AJ, Uthman OA, Spinewine A, Pham PA. Antiretroviral therapy adherence and drug–drug interactions in the aging HIV population. AIDS. 2012;26: S39–S53. 22781176 10.1097/QAD.0b013e32835584ea

[29] Malfertheiner P, Megraud F, O’Morain C, Bazzoli F, El-Omar E, Graham D, et al Current concepts in the management of *Helicobacter pylori* infection: the Maastricht III consensus report. Gut.2007;56:772–781. 17170018 10.1136/gut.2006.101634 PMC1954853

[30] Saruç M, Goksel G, Ozkaya S, Guclu F, Ozbakkaloglu B, Yuceyar H. The effect of CagA status on response to *Helicobacter pylori* eradication therapy in Western Turkey. Braz J Med Biol Res. 2001;34:1435–1439. 11668353 10.1590/s0100-879x2001001100010

[31] Figura N, Moretti E, Vaglio L, Langone F, Vernillo R, Vindigni C, et al Factors modulating the outcome of treatment for the eradication of *Helicobacter pylori* infection. New Microbiol. 2012;35:335–340. 22842603

[32] Houben M, Van de Beek D, Hensen E, de Craen AJ, Rauws EA, Tytgat GN. A systematic review of *helicobacter* eradication therapy. The impact of antimicrobial resistance on eradication rates. Aliment Pharmacol Ther. 1999;13:1047–1055. 10468680 10.1046/j.1365-2036.1999.00555.x

